# The Discovery and Characterization of a Potent DPP-IV Inhibitory Peptide from Oysters for the Treatment of Type 2 Diabetes Based on Computational and Experimental Studies

**DOI:** 10.3390/md22080361

**Published:** 2024-08-09

**Authors:** Zhongqin Chen, Xiaojie Su, Wenhong Cao, Mingtang Tan, Guoping Zhu, Jialong Gao, Longjian Zhou

**Affiliations:** 1 Shenzhen Institute of Guangdong Ocean University, Shenzhen 518120, China; chenzhongqin@gdou.edu.cn (Z.C.); 2112203108@stu.gdou.edu.cn (X.S.); cwenhong@gdou.edu.cn (W.C.); mttan@gdou.edu.cn (M.T.); 2 Guangdong Provincial Key Laboratory of Aquatic Products Processing and Safety, Guangdong Province Engineering Laboratory for Marine Biological Products, Zhanjiang Municipal Key Laboratory of Marine Drugs and Nutrition for Brain Health, National Research and Development Branch Center for Shellfish Processing (Zhanjiang), Guangdong Provincial Engineering Technology Research Center of Seafood, Guangdong Provincial Engineering Technology Research Center of Prefabricated Seafood Processing and Quality Control, College of Food Science and Technology, Guangdong Ocean University, Zhanjiang 524088, China; zjougp@gdou.edu.cn (G.Z.); gaojl@gdou.edu.cn (J.G.); 3 Collaborative Innovation Center of Seafood Deep Processing, Dalian Polytechnic University, Dalian 116034, China

**Keywords:** oyster peptides, DPP-IV, type 2 diabetes, network pharmacology, molecular docking, molecular dynamics simulation

## Abstract

The inhibition of dipeptidyl peptidase-IV (DPP-IV) is a promising approach for regulating the blood glucose levels in patients with type 2 diabetes (T2D). Oysters, rich in functional peptides, contain peptides capable of inhibiting DPP-IV activity. This study aims to identify the hypoglycemic peptides from oysters and investigate their potential anti-T2D targets and mechanisms. This research utilized virtual screening for the peptide selection, followed by in vitro DPP-IV activity assays to validate the chosen peptide. Network pharmacology was employed to identify the potential targets, GO terms, and KEGG pathways. Molecular docking and molecular dynamics simulations were used to provide virtual confirmation. The virtual screening identified LRGFGNPPT as the most promising peptide among the screened oyster peptides. The in vitro studies confirmed its inhibitory effect on DPP-IV activity. Network pharmacology revealed that LRGFGNPPT exerts an anti-T2D effect through multiple targets and signaling pathways. The key hub targets are AKT1, ACE, and REN. Additionally, the molecular docking results showed that LRGFGNPPT exhibited a strong binding affinity with targets like AKT1, ACE, and REN, which was further confirmed by the molecular dynamics simulations showcasing a stable peptide–target interaction. This study highlights the potential of LRGFGNPPT as a natural anti-T2D peptide, providing valuable insights for potential future pharmaceutical or dietary interventions in T2D management.

## 1. Introduction

Diabetes is a metabolic disorder characterized by insulin secretion defects or insulin resistance, caused by various factors such as genetics, diet, and environmental factors [1,2]. Its main clinical feature is high blood sugar, accompanied by disturbances in glucose and lipid metabolism, as well as amino acid metabolism. Currently, the incidence of diabetes is increasing yearly. According to the International Diabetes Federation (IDF), it is estimated that the number of global diabetes patients will reach 592 million by 2030 and 783.2 million by 2045. It is also projected that global healthcare spending related to diabetes will significantly increase from USD 966 billion in 2021 to USD 1054 billion in 2045 [3,4], making it a global public health issue. Diabetes can be classified into gestational diabetes (GD), type 1 diabetes (T1D), type 2 diabetes (T2D), and specific types of diabetes (monogenic diabetes, diseases of the exocrine pancreas, and drug- or chemical-induced diabetes) [5]. Amongst these, T2D predominates and affects more than 90–95% of the diabetic population [1,2]. Recently, the clinical drugs commonly used to treat T2D are mainly chemically synthesized, which have issues such as drug resistance, adverse reactions, and significant toxic side effects. On the other hand, natural hypoglycemic functional factors have a wide range of sources, low side effects, and affordable prices, making them highly prospective candidates for daily hypoglycemic care. It is good to note that, similar to synthetic compounds, natural products can also have undesired effects and require evaluation for safety and efficacy. Therefore, the development of natural active ingredients with anti-diabetic properties, after rigorous testing and validation, is of significant importance in expanding the therapeutic options for T2D [6,7].

Oyster is an aquatic shellfish with high economic, nutritional, and medicinal values. Oyster peptides are a group of important natural marine bioactive peptides with various effects such as antioxidant, immune-enhancing, liver-protective, and hypoglycemic activities [6,8]. The unique amino acid sequence structure of oyster bioactive peptides, derived from the particularity of the marine environment, confers them a natural advantage in reducing blood glucose [9]. Researchers have shown that peptides rich in branched-chain amino acids (BCAAs), including leucine, isoleucine, and valine, have effective dipeptidyl peptidase-IV (DPP-IV) inhibitory activity [8,9]. Therefore, these peptides have the potential to act as novel DPP-IV inhibitors, potentially leading to hypoglycemic effects on various tissue systems, such as the intestines, pancreas, liver, and stomach. In addition, BCAA-rich peptides not only exhibit good digestive stability for better physiological functionality but also promote the release of glucagon-like peptide-1 (GLP-1) and enhance the insulin response, thus regulating glucose homeostasis [10,11]. Hao et al. found that oyster peptides are abundant in BCAAs, and, when BCAAs are located at specific positions, such as the N-terminal end of the peptide chain, they exhibit significant DPP-IV inhibitory activity [8]. However, there is currently limited research on the hypoglycemic effects and mechanisms of marine-derived peptides (which is mainly focused on fish sources) [12]. Therefore, screening the hypoglycemic peptides from oyster bioactive peptides and exploring their mechanisms of action is necessary, which can offer a theoretical foundation and scientific direction for the further development and use of hypoglycemic peptides in treating or preventing T2D.

Network pharmacology is an interdisciplinary field that integrates theoretical methods from systems biology, structural biology, multiomics strategies, and other disciplines. It enables the analysis of the molecular mechanisms of the active substances in disease treatment from a systems and network perspective, incorporating information on active compounds, targets, and signaling pathways. This approach is beneficial for drug development and clinical applications [13,14,15]. Molecular docking and molecular dynamics simulations can provide valuable insights into the physical and chemical properties of bioactive molecules. Currently, they have been applied in drug screening, offering advantages such as increasing the discovery rate of drugs while reducing expensive experimental costs [16,17,18,19]. However, there is a limited number of studies that have applied molecular docking, molecular dynamics simulation, and network pharmacology strategies to identify potential hypoglycemic peptides and analyze their targets and mechanisms for treating T2D.

This study aimed to utilize the molecular docking, molecular dynamics simulation, and network pharmacology approaches to identify potent hypoglycemic peptides from oyster peptides and investigate their targets and mechanisms in T2D treatment, offering insights for the development, research, and clinical use of anti-T2D medications.

## 2. Results

### 2.1. Virtual Screening Identifies Potent DPP-IV Inhibitory Peptides from Oysters

Prior to virtual screening, a re-docking procedure was performed to validate the docking method, and the root mean square deviation (RMSD) between the obtained complexes and the resolved structures for DPP-IV was calculated. The analysis yielded an RMSD value of 0.52 Å, demonstrating the reliability of the docking approach, as RMSD values below 2 Å are generally considered indicative of a reliable docking method [20,21]. To obtain oyster peptides with a reasonable sequence and high affinity for T2D targets, this study employed DPP-IV as the target. Using the DPP-IV inhibitor linagliptin, approved by the Food and Drug Administration (FDA), as a positive control, molecular docking techniques were utilized to virtually screen 20 oyster peptides. The results showed that the binding affinity between the screened oyster peptide molecules and DPP-IV ranged from −6 to −9 (kcal/mol) (Table 1). Among them, the oyster peptide sequences SSGPIPTTPPPPPPVPK and LRGFGNPPT exhibited a lower binding affinity (−9 kcal/mol and −8.9 kcal/mol, respectively) with DPP-IV compared to the positive control Linagliptin (−8.7 kcal/mol), indicating that they had more stable binding conformations with DPP-IV and showed a good potential for anti-T2D activity. Furthermore, studies have shown that the hypoglycemic activity of peptides is most pronounced when the number of amino acid residues is less than 7, followed by peptides containing 7 to 25 amino acid residues, with the hypoglycemic activity decreasing as the chain length of the peptides increases [22,23] and shorter peptide sequences having lower immunogenicity and lower production costs in the later stages [24,25]. Therefore, the oyster peptide LRGFGNPPT was selected as the key peptide sequence for further research.

### 2.2. Synthesis and Validation of DPP-IV Inhibitory Activity of LRGFGNPPT

**Figure 1:**
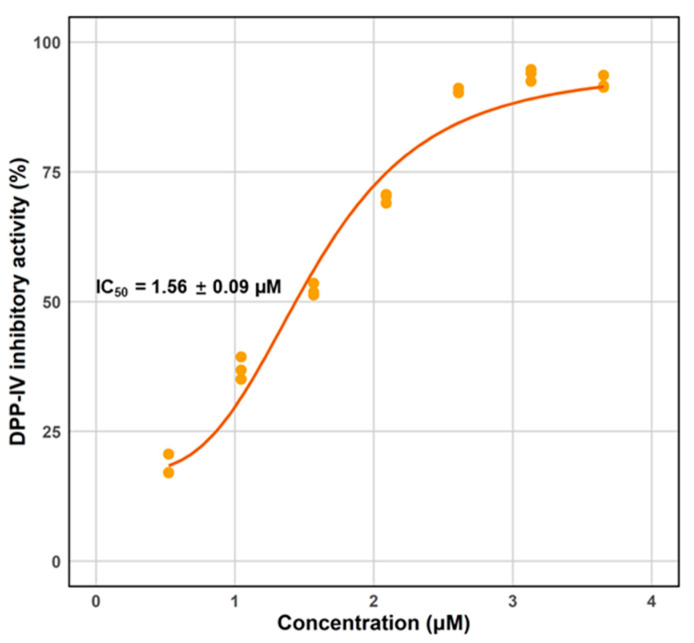
Inhibition of DPP-IV activity by peptide (LRGFGNPPT).

The oyster active peptide (LRGFGNPPT) was synthesized by the solid-phase synthesis method according to its amino acid sequence. The resulting synthetic peptide, LRGFGNPPT, exhibited a high purity of 98.13%, as validated by HPLC and LC-MS spectral analyses (Figure S1). As illustrated in Figure 1, the synthesized peptide LRGFGNPPT exhibited significant DPP-IV inhibitory activities, with a dose–response range from 0.52 to 3.65 μM and an IC_50_ value of 1.56 μM. Although the DPP-IV inhibitory activity of LRGFGNPPT is less potent compared to leading clinical drugs such as linagliptin (IC_50_ = 0.77 nM) [26], it is noteworthy within the context of food-derived peptides. The IC_50_ value of LRGFGNPPT compares favorably with other reported food-derived peptides, which have IC_50_ values ranging from 8.55 to 229.14 μM against DPP-IV [27,28,29]. The peptide’s natural origin and potential pharmacological benefits make it a promising candidate for further investigation for T2D.

### 2.3. Identification of Intersection Targets between LRGFGNPPT and T2D

**Figure 2:**
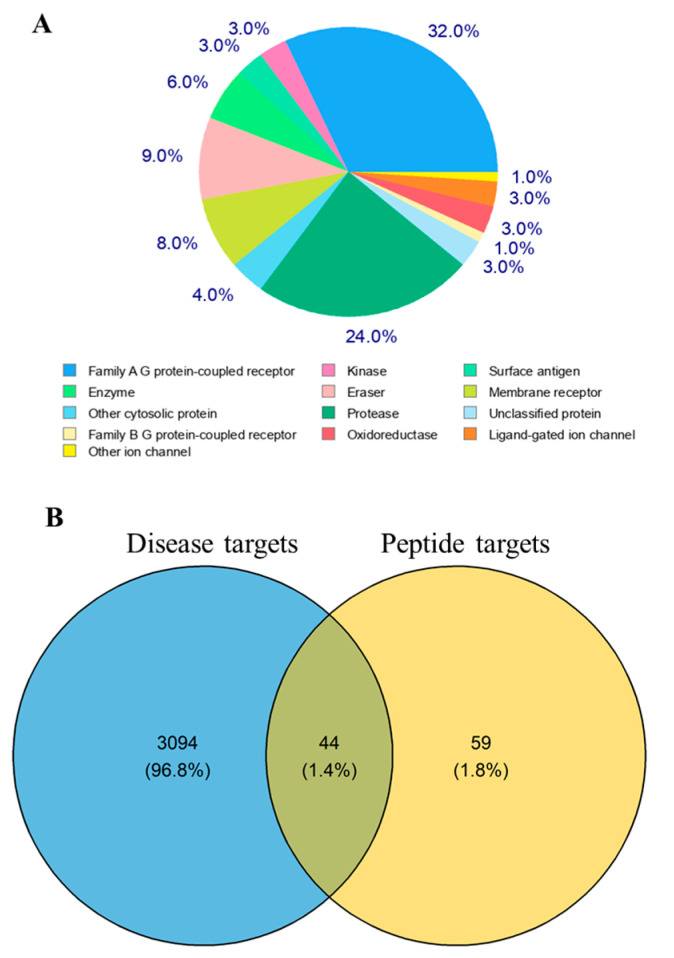
Target classification and T2D overlap of oyster active peptide (LRGFGNPPT): (**A**) classification of oyster active peptide (LRGFGNPPT) targets; and (**B**) overlapping targets between peptide (LRGFGNPPT) and T2D.

The potential targets of the oyster active peptide (LRGFGNPPT) were predicted using the Swiss TargetPrediction database [30], resulting in the identification of 103 targets. These targets were classified into 13 main categories, with the family A G protein-coupled receptor representing the highest proportion at 32% (Figure 2A). T2D-related genes were gathered from the DisGeNET database [31], GeneCards database [32], and Pharmacogenomics Knowledgebase [33], resulting in the identification of 3138 T2D-related targets. To obtain the relevant targets of the oyster active peptide (LRGFGNPPT) for anti-T2D activity, a Venn diagram was constructed to map the potential targets of the oyster active peptide (LRGFGNPPT) with the T2D-related targets, resulting in 44 overlap targets (Figure 2B).

### 2.4. PPI Network Analysis Reveals Core Targets of LRGFGNPPT in T2D Treatment

**Figure 3:**
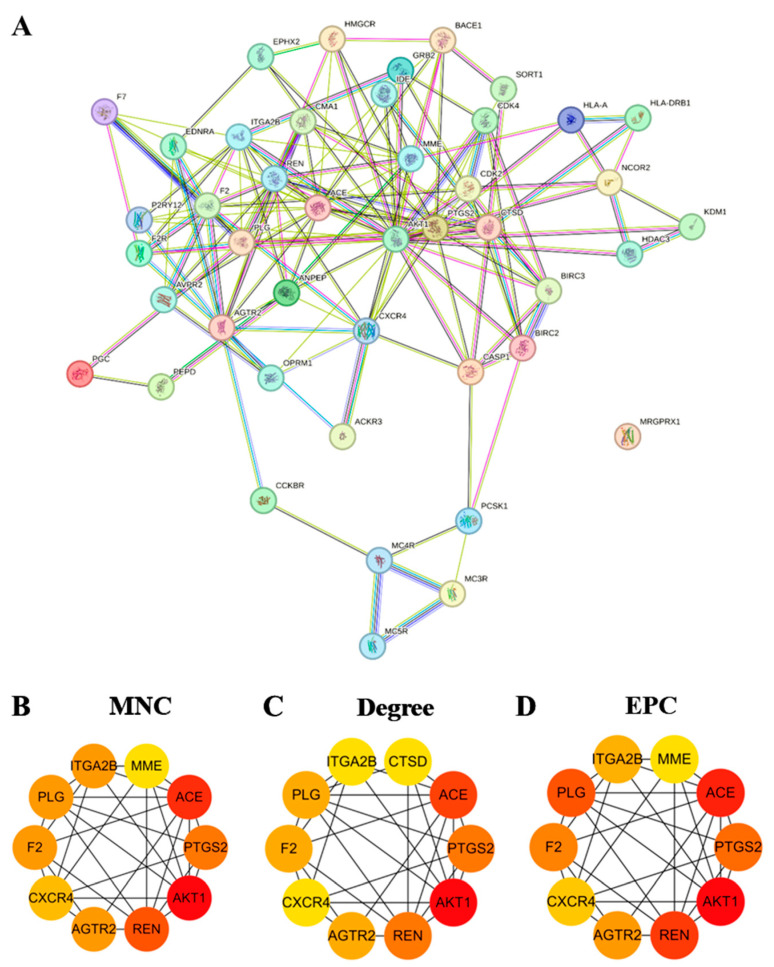
Network analysis of oyster active peptide’s anti-T2D mechanism. (**A**) The protein–protein interaction (PPI) network of oyster active peptide (LRGFGNPPT) with its anti-T2D targets: top 10 targets selected based on the three ranking methods; (**B**) maximum neighborhood component (MNC); (**C**) degree; and (**D**) edge percolated component (EPC). The intensity of the node’s color corresponds to its score.

In order to assess the impact of the intersection targets on T2D and examine their underlying associations, the 44 intersection targets underwent protein–protein interaction (PPI) network construction and analysis utilizing the STRING database and Cytoscape software. The findings are depicted in Figure 3A. Within the network diagram, the nodes correspond to targets, while the edges signify the interactions between the targets. This network comprises 44 action nodes and 150 interaction edges. Subsequently, the maximum neighborhood component (MNC), degree, and edge percolated component (EPC) methods were selected for further screening, with a focus on the top 10 targets identified from each method (Figure 3B–D). Notably, three hub targets—AKT1 (AKT Serine/Threonine Kinase 1), ACE (Angiotensin converting enzyme), and REN (Renin)—drew our specific attention as they consistently ranked high across all three algorithms. This consistently high ranking suggests that these targets may play crucial roles within the network, potentially making them key players associated with the peptide (LRGFGNPPT) for the treatment of T2D.

### 2.5. Enrichment Analysis of LRGFGNPPT Target Pathways for Anti-T2D

**Figure 4:**
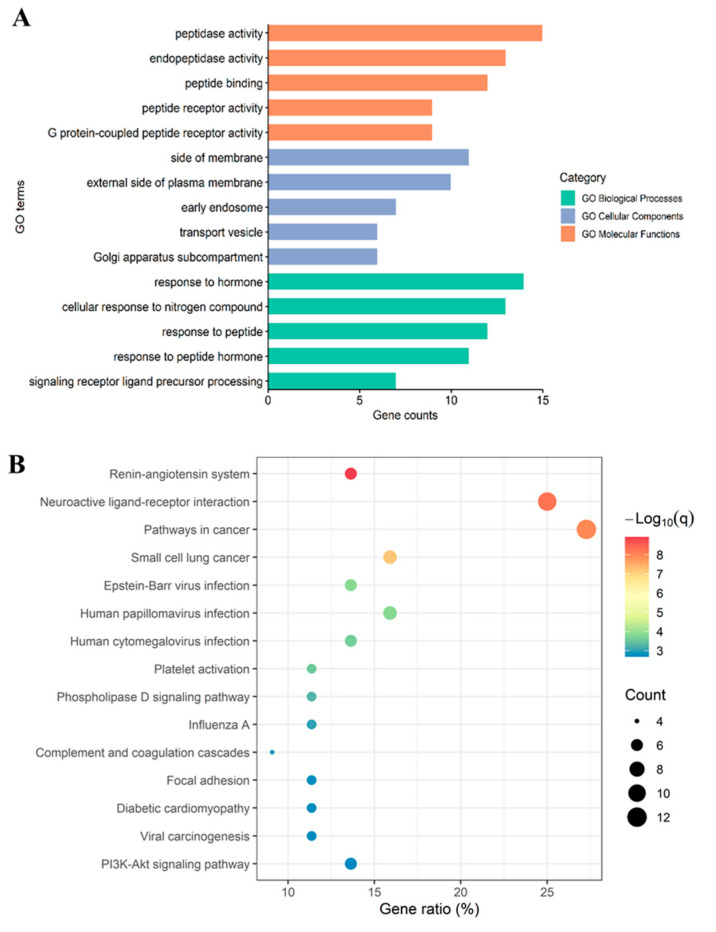
Enrichment analysis based on the common genes between peptide (LRGFGNPPT) and T2D: (**A**) GO analysis; and (**B**) KEGG pathway analysis.

To further investigate the potential pharmacological mechanisms of the active oyster peptide LRGFGNPPT in anti-T2D activity, enrichment analysis (GO and KEGG analyses) was performed on the 44 intersection targets. The GO analysis was categorized into Biological Processes (BPs), Cellular Components (CCs), and Molecular Functions (MFs). In this study, the GO analysis revealed 332 enriched pathways for BPs, 34 enriched pathways for CCs, and 30 enriched pathways for MFs. Figure 4A shows the top five items for BPs, CCs, and MFs. The highly enriched GO items in the BPs were the signaling receptor ligand precursor processing, response to peptide, response to hormone, and others. In terms of CCs, the highly enriched GO items were the external side of the plasma membrane, the side of the membrane and an early endosome. In the MFs, the highly enriched GO items were the peptidase activity, endopeptidase activity, and peptide binding. These processes are intimately linked with the pathogenesis and complications of T2D [34,35,36]. These results indicated that the oyster active peptide LRGFGNPPT exerts its anti-T2D effects by participating in multiple biological processes, affecting various cellular components and molecular functions.

A KEGG analysis was performed on the 44 intersection targets, resulting in a total of 59 enriched signaling pathways. The top 15 pathways are shown in Figure 4B. The results indicated that the targets were enriched in the renin–angiotensin system, pathways in cancer, diabetic cardiomyopathy, PI3K–Akt signaling pathway, and others. These findings suggested that T2D is a metabolic disorder with complex mechanisms regulated by multiple pathways and targets, and the oyster active peptide LRGFGNPPT may exert its anti-T2D effects through these signaling pathways. Furthermore, the results showed that cancer-related pathways rank high, which may be due to the common mechanisms shared between T2D and cancer [37,38].

### 2.6. Molecular Docking Reveals a High Affinity between LRGFGNPPT and Its Core Anti-T2D Targets

**Figure 5:**
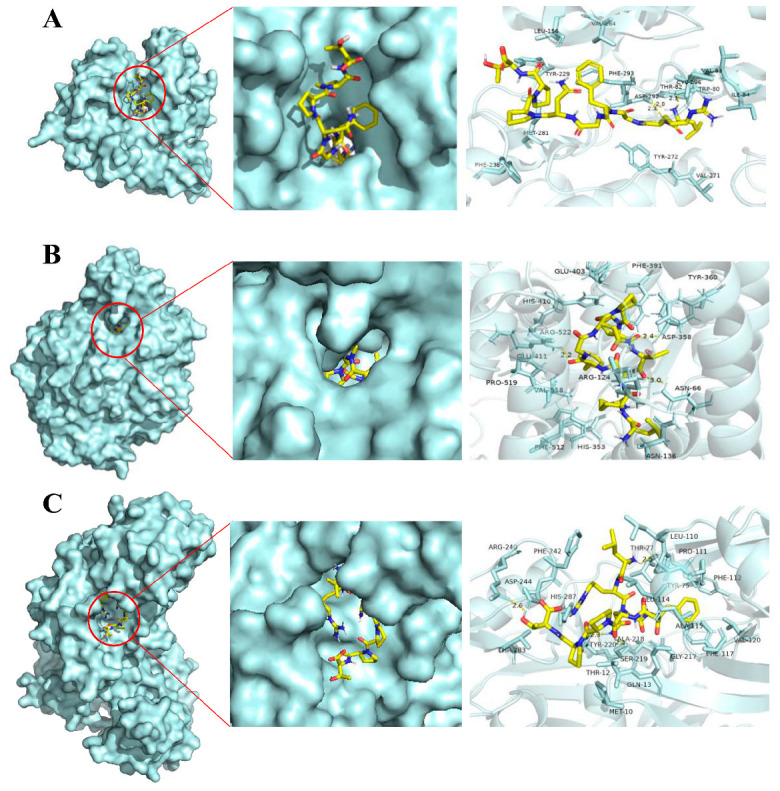
Molecular docking of peptide (LRGFGNPPT) with its potential targets. Three-dimensional maps between peptide (LRGFGNPPT) and AKT1 (**A**), ACE (**B**), or REN (**C**).

To validate the docking method, the co-crystallized ligands were re-docked, and the RMSD between the obtained complexes and the resolved structures for AKT1, ACE, and REN was calculated. RMSD values of 0.98 Å, 0.63 Å, and 1.05 Å were obtained, respectively, indicating the reliability of the docking method (RMSD < 2 Å) [20,21]. To further elucidate the potential interactions between the active oyster peptide LRGFGNPPT and its core anti-T2D targets (AKT1, ACE, and REN), the molecular docking study was conducted. The results (Table 2) showed that the binding energies between LRGFGNPPT and the targets AKT1, ACE, and REN were −9.3, −8.9, and −9.0 kcal/mol, respectively, indicating a strong interaction between LRGFGNPPT and the targets. Furthermore, based on the affinity obtained from the molecular docking study, AKT1 emerged as the target with the most favorable interaction with LRGFGNPPT. Specifically, the affinity between LRGFGNPPT and AKT1 was −9.3 kcal/mol, which was the lowest among the three targets: AKT1 (−9.3 kcal/mol), ACE (−8.9 kcal/mol), and REN (−9.0 kcal/mol). An analysis of the intermolecular forces revealed that the interactions between LRGFGNPPT and the targets primarily involved hydrogen bonds, hydrophobic forces, and others (Figure 5). Amongst these, LRGFGNPPT interacted with AKT1 by forming hydrogen bonds with Thr82 and Asp292, while also engaging in hydrophobic interactions with neighboring amino acid residues, such as Phe293 and Cys296 (Figure 5A). Similar interactions were observed between LRGFGNPPT and ACE (Figure 5B) or REN (Figure 5C). In summary, the active oyster peptide LRGFGNPPT exhibited a high affinity for its core targets AKT1, ACE and REN and interacted with these targets through specific amino acid residue sites. These interactions may play a key role in its anti-T2D effects.

### 2.7. Molecular Dynamics Simulation Confirms Stable Interaction of LRGFGNPPT with AKT1

**Figure 6:**
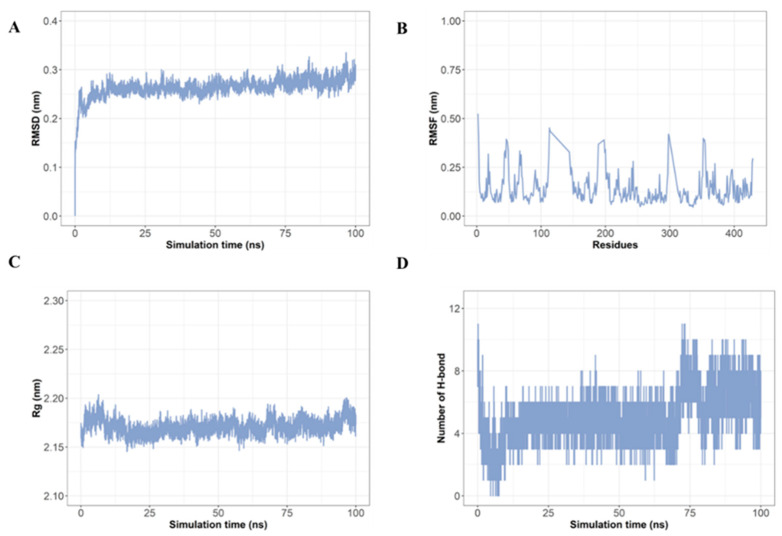
Molecular dynamics simulation of peptide (LRGFGNPPT) with AKT1: (**A**) RMSD analysis; (**B**) RMSF analysis; (**C**) gyration radius; and (**D**) hydrogen bonds.

Based on the binding energy analysis, AKT1 exhibited the most favorable interaction with LRGFGNPPT, making it the optimal target. Therefore, we employed a molecular dynamics (MD) simulation to evaluate the stability of the LRGFGNPPT–AKT1 complex to gain a deeper understanding of its potential mechanisms. Specifically, we conducted an extensive 100 ns MD simulation experiment to thoroughly investigate the dynamic properties and stability of this complex (Figure 6). Initially, we utilized root mean square deviation (RMSD) as a key metric to assess the overall conformational stability of the system. The results revealed that the protein–ligand complex reached a stable conformational convergence around 12 ns during the 100 ns simulation period and maintained a high stability throughout the subsequent simulation period (Figure 6A). This strongly suggested that the complex had entered into a stable conformational state. Subsequently, the flexibility changes in the individual residues of the AKT1 protein during the simulation process were evaluated by analyzing the root mean square fluctuations (RMSFs). The data showed that the RMSF values for the AKT1 protein residues in contact with LRGFGNPPT primarily ranged between 0.05 and 0.55 nm, exhibiting a generally mild and slight fluctuation (Figure 6B), indicating a stable binding between the ligand and the protein. Furthermore, the global structural compactness of the protein during the simulation was assessed by calculating the radius of gyration (Rg). As illustrated in Figure 6C, the complex demonstrated a relatively small radius of gyration, suggesting a high structural stability for the complex. Moreover, the number and stability of the hydrogen bonds play a critical role in the interactions between the protein and ligand [39,40]. The results showed that, within the 100 ns timescale, the number of hydrogen bonds maintained between LRGFGNPPT and AKT1 primarily ranged from 2 to 10, with a notable increase observed in the latter stages of the simulation (Figure 6D), further reinforcing the robustness of their binding interaction. In summary, these findings indicated that LRGFGNPPT is capable of forming a stable complex structure with AKT1 and potentially exerting its inherent biological activity.

### 2.8. Analysis of ADMET Properties and Susceptibility to Degradation by Proteases of LRGFGNPPT

To predict the ADMET (absorption, distribution, metabolism, excretion, and toxicity) properties and susceptibility to degradation by the proteases of LRGFGNPPT, ADMETlab 3.0 [41] and PeptideCutter [42] were used. The corresponding results were displayed in Tables S1–S3. The results indicated that LRGFGNPPT has generally acceptable physicochemical and ADMET properties (Tables S1 and S2), including favorable plasma protein binding (14.045) and plasma clearance (2.205) and low toxicity profiles, such as drug-induced liver injuries (DILI: 0.038), AMES toxicity (0.031), and rat oral acute toxicity (0.025). Additionally, LRGFGNPPT exhibits no significant interaction with major cytochrome P450 enzymes, indicating a low risk of metabolic interactions (Table 3). While LRGFGNPPT showed some limitations, such as less than ideal absorption, these aspects can be addressed through further optimization. The analysis using PeptideCutter indicates that LRGFGNPPT is susceptible to degradation by proteases, specifically by Chymotrypsin (both high and low specificity), Pepsin (at a pH of 1.3 and above 2), and Trypsin (Table S3). Despite this susceptibility, the peptide’s natural food origin, comparative potency, and favorable safety profile make it a promising candidate for further exploration.

## 3. Discussion

The inhibition of DPP-IV is a promising strategy to prevent and treat T2D [43,44]. In this study, DPP-IV was selected as the target, and virtual molecular docking was conducted on 20 oyster peptides. The virtual screening approach effectively identified the peptides from oysters, particularly LRGFGNPPT, with the potential to be natural DPP-IV inhibitors. This finding is consistent with the known functionality of food-derived peptides in metabolic regulation and adds a novel candidate to the repertoire of potential anti-T2D agents [43,44,45]. The solid-phase synthesis and subsequent validation of the peptide LRGFGNPPT showed high purity and a potent inhibition of DPP-IV activity. Although the DPP-IV inhibitory activity of LRGFGNPPT is less potent compared to linagliptin [26], it stands out among the food-derived peptides. The IC_50_ value of LRGFGNPPT (1.56 µM) compares favorably with other food-derived peptides, such as LSICGEESFGTGSDHIR, SLGESLLQEDVEAHK, and QLRDIVDK from Sorghum bicolor (IC_50_ values of 73.5, 82.5, and 8.55 µM, respectively) [27]; LPIIDI and APGPAG from silver carp proteins (IC_50_ values of 105.44 and 229.14 μM, respectively) [28]; and RLYLHENK and MQEHFTCCR from sheep (IC_50_ values of 166.4 and 214.8 μM, respectively) [29]. These comparisons highlight the potential of LRGFGNPPT as a natural food-derived DPP-IV inhibitor, offering a promising avenue for the development of novel therapeutic agents for T2D. This outcome aligns with our initial in silico predictions. Unlike synthetic drugs, however, LRGFGNPPT offers the advantage of being a naturally derived peptide, which may translate to fewer adverse effects and better patient compliance.

In targeting DPP-IV, we aimed to prolong the activity of glucagon-like peptide-1 (GLP-1), a peptide hormone secreted by the intestinal L cells. GLP-1 has several effects: (1) it can promote insulin secretion by pancreatic beta cells; (2) it can reduce the alpha cell response, inhibiting glucagon secretion; (3) it can decrease the liver glucose output; and (4) it can help delay gastric emptying, promoting a feeling of satiety and reducing appetite, with a low risk of hypoglycemia. Thus, GLP-1 can systemically regulate blood glucose levels at multiple levels and is an advantageous target for the treatment of T2D [46,47]. However, GLP-1 is often rapidly degraded by DPP-IV in the body, resulting in an approximately 95% loss of activity and a very short half-life. Therefore, inhibiting DPP-IV activity to delay GLP-1 degradation is an important strategy for the development of T2D drugs. The oyster peptide LRGFGNPPT exhibited potent DPP-IV inhibitory activity and has great potential as an anti-T2D agent. By inhibiting DPP-IV, LRGFGNPPT may help maintain higher GLP-1 levels, promoting insulin secretion, reducing glucagon release, decreasing hepatic glucose production, and slowing gastric emptying. This multifaceted impact on glucose regulation underscores the therapeutic value of DPP-IV inhibitors for T2D treatment.

According to previous studies, the specific structure of the active peptides, including the amino acid composition, hydrophobicity, sequence, and chain length, determines their biological activity. Generally, the peptides with a higher proportion of hydrophobic amino acids could exhibit higher activity [48]. The hypoglycemic effect of hypoglycemic peptides depends on their structure, typically composed of 2–25 amino acid residues, with molecular weights ranging from 228 to 3000 Da. The amino acid residues leucine (Leu), arginine (Arg), glutamate (Glu), phenylalanine (Phe), proline (Pro), and alanine (Ala) in the peptide sequence contribute to their hypoglycemic activity [49]. Furthermore, when hydrophobic amino acids or Pro are located in the first four positions at the N-terminus of the peptide, with Pro being near the C-terminus and the branched-chain amino acids (BCAAs), such as Val, Leu, and Ile, being placed within the first three positions at the N-terminus, the peptide often exhibits significant DPP-IV inhibitory activity and has great hypoglycemic potential [50]. In this study, the screened oyster peptide LRGFGNPPT consists of nine amino acids, with a molecular weight of 957.5 Da. It has a high hydrophobicity ratio of 44.4%, indicating favorable hydrophobic properties. Moreover, it has the distinct structural features of hypoglycemic peptides, with leucine at the first position of the N-terminus and two proline residues near the C-terminus. These characteristics make it suitable for anti-T2D applications and show promising prospects.

A protein–protein interaction network was constructed for the intersection targets of LRGFGNPPT and T2D, consisting of 44 targets. The top three ranking targets were AKT1, ACE, and REN based on the three ranking methods—MNC, Degree and EPC—indicating that these three targets are the main targets of LRGFGNPPT in its anti-T2D effects. AKT1, an important protein kinase, participates in various cellular processes, including apoptosis and glucose metabolism, and plays a crucial role in the treatment of T2D and its complications [51,52]. AKT1 is one of the subtypes of AKT (AKT1, AKT2, and AKT3), and AKT is an important downstream component of the phosphatidylinositol 3-kinase/protein kinase B signaling pathway (PI3K–AKT pathway). It exerts therapeutic effects in T2D, obesity, and cancer by regulating insulin resistance, lipid metabolism, and cell apoptosis [53,54]. ACE is an essential enzyme involved in the renin–angiotensin system (RAS), which regulates blood pressure and fluid balance. In addition to its traditional role in cardiovascular health, ACE contributes significantly to the pathophysiology of T2D and its complications [55]. As part of the RAS, ACE converts angiotensin I to the potent vasoconstrictor angiotensin II, which can lead to increased oxidative stress and inflammation, exacerbating insulin resistance and beta cell dysfunction [56,57]. Moreover, ACE inhibitors are widely used to mitigate these adverse effects, offering therapeutic benefits in the management of T2D by improving glycemic control and protecting against T2D-induced kidney and vascular damage [58,59]. REN, a key enzyme in the renin–angiotensin system (RAS), initiates the conversion of angiotensinogen to angiotensin I. Its elevated activity in T2D contributes to increased oxidative stress and inflammation, exacerbating insulin resistance and beta cell dysfunction [60,61]. Renin inhibitors, such as Aliskiren, offer therapeutic benefits by reducing these adverse effects and improving glycemic control, thereby protecting against T2D-induced kidney and vascular damage [62]. In summary, targeting AKT1, ACE, and REN presents a promising strategy for managing T2D.

The GO and KEGG enrichment analyses provided further insights into the molecular mechanisms by which LRGFGNPPT exerts its effects. The key BPs, such as receptor ligand precursor processing, peptide response, and hormone response, align with T2D pathogenesis and complications [63,64]. The CC findings related to membrane components and endosomes highlight the importance of cellular signaling in glucose metabolism [65]. In the MFs, the peptidase and endopeptidase activities suggest the enzymatic regulation of bioactive peptides. The KEGG pathway analysis identified the top 15 factors, including the renin–angiotensin system, pathways involved in cancer, diabetic cardiomyopathy, and the PI3K–Akt signaling pathway. The enrichment of the renin–angiotensin system aligns with the existing literature on the significant role of this pathway in regulating blood pressure and fluid balance in T2D patients [66,67]. The PI3K–Akt signaling pathway is well documented for its central role in insulin signaling and glucose homeostasis [68,69], indicating that LRGFGNPPT may exert its anti-T2D effects by modulating this critical pathway. The presence of cancer-related pathways among the top results is particularly noteworthy. Recent studies have highlighted the shared mechanisms between T2D and cancer, such as inflammation, oxidative stress, and a dysregulated cellular metabolism [37,38]. The involvement of the peptide in various biological processes and pathways reflects the intricate nature of T2D as a multifaceted metabolic disorder. The multi-target approach of LRGFGNPPT has the potential to provide comprehensive benefits by regulating a range of biological processes and signaling pathways, thereby offering therapeutic possibilities for T2D and its associated complications.

In this study, the molecular docking between LRGFGNPPT and the top three core targets AKT1, ACE, and REN was conducted. In the molecular docking results, it is generally considered that an affinity less than 0 indicates spontaneous binding between the ligand and receptor, and the lower the affinity, the higher the likelihood of interaction [70]. The molecular docking results revealed that LRGFGNPPT has a high affinity for AKT1, ACE, and REN, involving hydrophobic interactions and hydrogen bonds with key amino acid residues. These findings suggested that LRGFGNPPT may exert its anti-T2D effects through these targets.

Moving to the molecular dynamics simulation, the stability of the LRGFGNPPT–AKT1 complex was rigorously tested over a 100 ns simulation period. The RMSD analysis confirmed that the complex swiftly reached and maintained a stable conformation, suggesting a robust binding configuration. Complementary RMSF analysis revealed minimal fluctuations in the residues interacting with LRGFGNPPT, supporting a stable interaction. The compactness of the complex, as measured by the radius of gyration, also pointed towards a structurally tight and stable complex. Notably, the consistent presence of hydrogen bonds, even increasing over time, underscores the durability and strength of the ligand–protein association.

The ADMET properties of LRGFGNPPT, evaluated using ADMETlab 3.0 [41], showed generally acceptable physicochemical and ADMET characteristics, including favorable plasma protein binding and plasma clearance and low toxicity profiles. It exhibited no significant interaction with the major cytochrome P450 enzymes, suggesting a low risk of metabolic interactions. Although the PeptideCutter analysis indicated a susceptibility to degradation by peptidases like Chymotrypsin, Pepsin, and Trypsin, the peptide’s natural origin and favorable safety profile make it a promising candidate for further research. Future studies should aim to optimize its stability and pharmacokinetics through strategies such as chemical modification, cyclization, and peptidomimetics to enhance its resistance to enzymatic degradation while maintaining or enhancing its DPP-IV inhibitory activity.

Given these promising results, future research should explore the in vivo efficacy and safety of LRGFGNPPT. Clinical studies will be essential to confirm its potential as a dietary supplement or pharmaceutical agent. Additionally, structure–activity relationship (SAR) studies could lead to the development of more potent analogs of LRGFGNPPT, further enhancing its anti-T2D efficacy.

## 4. Materials and Methods

The oyster peptides used in the screening were obtained from our previous research [71]. The DPP-IV inhibitor screening assay kit was obtained from Cayman Chemical Co. (Ann Arbor, MI, USA). All the other chemicals used in this study were of analytical grade.

### 4.1. Virtual Screening of Oyster DPP-IV Inhibitory Peptides

To obtain oyster peptides with reasonable sequences and a high affinity for T2D targets, this study used DPP-IV as the target and its inhibitor Linagliptin as the positive control. Twenty oyster peptides were virtually screened using molecular docking technology. First, the 3D structures of the oyster peptides were generated using an RDKit (https://www.rdkit.org/ accessed on 1 March 2024) to convert the peptide sequence information to SMILES, followed by energy minimization and conversion to the mol2 file format using OpenBabel 3.1.1 [72]. Then, the hydrogens were added, Gasteiger charges were assigned, and the number of torsions were established during the transformation to pdbqt files with AutoDockTools 1.5.6 [73]. The ligands in pdbqt format were used for further virtual screening through AutoDock Vina 1.2.0. The RCSB PDB database (http://www.rcsb.org/ accessed on 1 March 2024) was utilized to acquire the DPP-IV structure of the T2D target (PDB accession number: 3KWF). The PyMol software (https://www.PyMOL.org accessed on 1 March 2024) was used for the initial visualization and basic processing of the DPP-IV structure, which involved removing the water molecules and separating the original ligand. Following this, AutoDockTools 1.5.6 was used for the preparation of the protein structures for docking, including adding hydrogens and calculating the Gasteiger charges. The final protein structures were saved in the pdbqt format required by AutoDock Vina 1.2.0. The grid box was set by Autodocktools 1.5.6 based on the original ligands. For DPP-IV, the search space coordinates XYZ were 57.292 × 62.902 × 53.042, the dimension XYZ was 38 × 40 × 76, and the spacing was 1 Å. Subsequently, AutoDock Vina 1.2.0 [74,75] was employed for the semi-flexible molecular docking of the oyster peptides with DPP-IV, resulting in an affinity between the oyster peptides and DPP-IV. Based on the rank of affinity, the oyster peptides with reasonable peptide sequence lengths (amino acid (AA) sequence length <15 AA) and a high affinity were selected for further research.

To validate the reliability of the molecular docking protocol, we performed the re-docking of the known ligands into their respective binding sites of the protein using AutoDock Vina 1.2.0. The RMSD values between the docked complex and the resolved structure were calculated using PyMOL.

### 4.2. Peptide Synthesis and Analysis of Its DPP-IV Inhibitory Activity In Vitro

Based on the results of the virtual screening, the optimal oyster peptide (key peptide sequences with potent DPP-IV inhibition) was selected and synthesized by Wuxi Maimertop Biotechnology Co., Ltd. (Wuxi, China). Then, the synthesized peptide was subjected to the DPP-IV inhibition activity verification. The DPP-IV inhibitory activity was determined following the instructions of commercial kits: a DPP (IV) inhibitor screening assay kit (700210 Cayman chemicals, USA). Briefly, 30 µL of assay buffer, 10 µL of DPP-IV enzyme, and 10 µL of peptide solution were added to a 96-well plate. Then, 50 µL of substrate (Gly-Pro-AMC, Sigma-Aldrich, Saint Louis, MO, USA) was added to start the reaction. After incubation at 37 °C for 30 min, the absorbance of the reaction mixture was detected under excitation and emission wavelengths of 355 nm and 460 nm, respectively. The DPP-IV inhibitory activity was calculated using the following formula:

(1) $$\text{DPP-IV}\;\mathrm{Inhibition}\;(\%)=\frac{A-\;B}{A}\times 100\%$$

where A was the average fluorescence of 100% initial activity (blank) and B was the fluorescence of the sample.

The 50% inhibitory concentration (IC_50_) of DPP-IV enzyme activity was calculated using the drc package (https://cran.r-project.org/web/packages/drc/ accessed on 20 July 2024) in R 4.1.2.

### 4.3. Potential Targets Prediction of Oyster Peptide and T2D

Based on the results of the virtual screening, the optimal oyster peptides (key peptide sequences with potent DPP-IV inhibition) were selected. The RDkit was used to obtain their structures and SMILE encoding. The Swiss Target Prediction database (http://www.swisstargetprediction.ch/) [30] accessed on 15 March 2024 was used to predict the targets of the key peptide sequences. This study collected the T2D-associated genes by utilizing the DisGeNET database (https://www.disgenet.org/) [31] accessed on 15 March 2024, with supplementary targets pertaining to T2D sourced from the GeneCards database (https://www.genecards.org) [32] accessed on 15 March 2024 and the Pharmacogenomics Knowledgebase (https://www.pharmgkb.org) [33] accessed on 15 March 2024. To obtain key targets for the anti-T2D activity of the key peptide sequences, the ggvenn package in R software was used to draw a Venn diagram, intersecting the potential targets of the key peptide sequences with the T2D targets, thus identifying the intersection targets between the two.

### 4.4. Protein–Protein Interaction (PPI) Network Construction and Core Targets Identification

The intersection targets were uploaded to the STRING database (https://string-db.org/) [76] accessed on 20 March 2024, and the “Multiple Proteins” option was set to “Homo sapiens” to construct the protein–protein interaction (PPI) network. The raw data of the protein interaction network were exported and imported into Cytoscape 3.7.2 software. The maximum neighborhood component (MNC), degree, and edge percolated component (EPC) algorithm in CytoHubba was used for an in-depth analysis of the data and the top 10 core targets were obtained.

### 4.5. Enrichment Analysis

Metascape [77] was used to perform the gene ontology (GO) and Kyoto Encyclopedia of Genes and Genomes (KEGG) enrichment analyses on the intersection targets of the oyster key peptide sequences and T2D. A GO analysis includes three aspects: biological process (BP), cellular component (CC), and molecular function (MF). The top 15 enrichment results were selected for each category based on *p*-values (*p* < 0.01). The R software was used for the visualization, providing a graphical representation of the main biological functions and related pathways involved in the anti-T2D effects of the oyster peptide, thus deciphering their underlying mechanisms.

### 4.6. Molecular Docking Analysis

To further investigate the affinity and binding sites between the key oyster peptide sequences and their core anti-T2D targets (the top three ranked), molecular docking technology (AutoDock Vina 1.2.0) was employed [74,75]. First, the 3D structures of the key peptide sequences were generated by using the same protocols described in Section 4.1, and the RCSB PDB database (http://www.rcsb.org/) accessed on 1 March 2024 was utilized to acquire the structures of the core anti-T2D targets. The software PyMol (https://www.PyMOL.org) accessed on 1 March 2024 was used for the initial visualization and basic preprocessing of the core targets (removing the water molecules and separating the original ligands). Subsequently, AutoDockTools 1.5.6 was used for the preparation of the protein structures for docking. The final protein structures were saved in the PDBQT format required by AutoDock Vina 1.2.0. The grid box was set by Autodocktools 1.5.6 based on the original ligands. For AKT1, the search space coordinates XYZ were 6.286 × −7.919 × 17.221, the dimension XYZ was 36 × 28 × 28, and the spacing was 1 Å. For ACE, the search space coordinates XYZ were 40.617 × 37.334 × 43.393, the dimension XYZ was 26 × 28 × 40, and the spacing was 1 Å. For REN, the search space coordinates XYZ were 23.384 × 29.420 × 75.172, the dimension XYZ was 42 × 46 × 40, and the spacing was 1 Å. Following this, AutoDock Vina 1.2.0 was employed for the semi-flexible molecular docking. The results were visualized using PyMol.

### 4.7. Molecular Dynamics Simulation

To investigate the stability of the docked protein–ligand complex, we conducted a 100ns molecular dynamics simulation with Gromacs2022 [78]. We utilized the AMBER14SB force field [79] and incorporated water molecules based on the TIP3P water model, and the system charges were neutralized with sodium or chloride ions. Subsequently, the system underwent equilibration in the canonical ensemble (NVT) and the isothermal–isobaric ensemble (NPT), maintaining the ambient temperature and pressure conditions, and was then subjected to a 100 ns molecular dynamics simulation. Upon completion of the simulations, we analyzed the trajectories using various metrics, including the root mean square deviation (RMSD), root mean square fluctuations (RMSFs), radius of gyration (Rg), and hydrogen bond, to assess the stability and dynamic behavior of the protein–ligand complex.

### 4.8. Prediction of ADMET Properties and Susceptibility to Degradation by Proteases

To evaluate the pharmacokinetic profile and potential toxicity of the oyster peptide, we employed ADMETlab 3.0 (https://admetlab3.scbdd.com/server/screening) accessed on 21 July 2024, a comprehensive online platform for predicting the ADMET (absorption, distribution, metabolism, excretion, and toxicity) and drug-likeness properties of compounds [41]. To assess the potential susceptibility of the oyster peptide to degradation by proteases, we utilized PeptideCutter (https://web.expasy.org/peptide_cutter/) accessed on 21 July 2024, a comprehensive online tool for predicting the potential cleavage sites of peptides by various proteases [42].

## 5. Conclusions

In conclusion, the peptide LRGFGNPPT from oysters has emerged as a promising candidate for managing T2D through DPP-IV inhibition and multi-target modulation. This study highlights the potential of marine-derived peptides in therapeutic applications and opens new avenues for the development of natural anti-T2D agents. Future studies should focus on optimizing its stability and pharmacokinetic properties to maximize its therapeutic potential for T2D. Further in vivo studies will be essential to fully evaluate its safety and efficacy in preclinical settings.

## Figures and Tables

**Table 1:** Results of a virtual screening of oyster peptides using DPP-IV as the target.

Target	PDB Entry	Ligand	Affinity (kcal/mol)
DPP-IV	3KWF	Linagliptin (positive control)	−8.7
DPP-IV	3KWF	SSGPIPTTPPPPPPVPK	−9
DPP-IV	3KWF	LRGFGNPPT	−8.9
DPP-IV	3KWF	YDDTYVPR	−8.4
DPP-IV	3KWF	GEDGAEGPTGPVGPL	−8
DPP-IV	3KWF	NGEVGPLGLPG	−8
DPP-IV	3KWF	YDNLPAECKLA	−8
DPP-IV	3KWF	GEPGPEGPAGPIGPR	−7.9
DPP-IV	3KWF	QDRDHIIIGWEP	−7.8
DPP-IV	3KWF	IDEDIEPPR	−7.7
DPP-IV	3KWF	VDVVLPK	−7.7
DPP-IV	3KWF	GPSGEPGPEGPAGPIGPR	−7.6
DPP-IV	3KWF	EAAKGGGETWILYRG	−7.6
DPP-IV	3KWF	GVGDDIAPR	−7.6
DPP-IV	3KWF	LPYDKPGAPGTPK	−7.5
DPP-IV	3KWF	GLIDEDIEPPR	−7.4
DPP-IV	3KWF	LVLECKASNPH	−7.4
DPP-IV	3KWF	QDIGGQIPGNKGQN	−7.2
DPP-IV	3KWF	QEAEVFSIMENL	−7.1
DPP-IV	3KWF	DMEGKPSPPGPS	−6.9
DPP-IV	3KWF	ITTLLTAI	−6.5

**Table 2:** Molecular docking affinity (kcal/mol) of AKT1, ACE, and REN with LRGFGNPPT.

Protein	PDB Entry	Ligand	Affinity (kcal/mol)
AKT1	3o96	LRGFGNPPT	−9.3
ACE	1o8a	LRGFGNPPT	−8.9
REN	2v0z	LRGFGNPPT	−9.0

**Table 3:** The representative properties of LRGFGNPPT.

Property	Value	Decision
Pfizer rule	Acceptable	Excellent
Caco-2 permeability	−6.639	Bad
Plasma protein binding (PPB)	14.045	Excellent
Plasma clearance	2.205	Excellent
The half-life (T_1/2_)	1.18	Medium
Drug-induced liver injury (DILI)	0.038	Excellent
AMES toxicity	0.031	Excellent
Rat oral acute toxicity	0.025	Excellent
CYP1A2 inhibitor	0.0	Excellent
CYP1A2 substrate	0.0	Excellent

## Data Availability

Data will be made available on request.

## Supplementary Materials

The following supporting information can be downloaded at https://www.mdpi.com/article/10.3390/md22080361/s1, Figure S1: HPLC and LC-MS spectrum of the synthetic peptide (LRGFGNPPT). Table S1. Physicochemical properties of LRGFGNPPT through ADMETlab 3.0. Table S2. ADMET properties of LRGFGNPPT through ADMETlab 3.0. Table S3. Prediction of potential cleavage sites by various proteases for LRGFGNPPT using PeptideCutter.
