# Supplementary material for: The Discovery and Characterization of a Potent DPP-IV Inhibitory Peptide from Oysters for the Treatment of Type 2 Diabetes Based on Computational and Experimental Studies
Source: Mar Drugs. 2024 Aug 9;22(8):361. doi: 10.3390/md22080361 (PMC11355449; doi:10.3390/md22080361)
Supplement: Supplementary file 1 [file marinedrugs-22-00361-s001.zip › marinedrugs-3119459-supplementary.pdf]

A

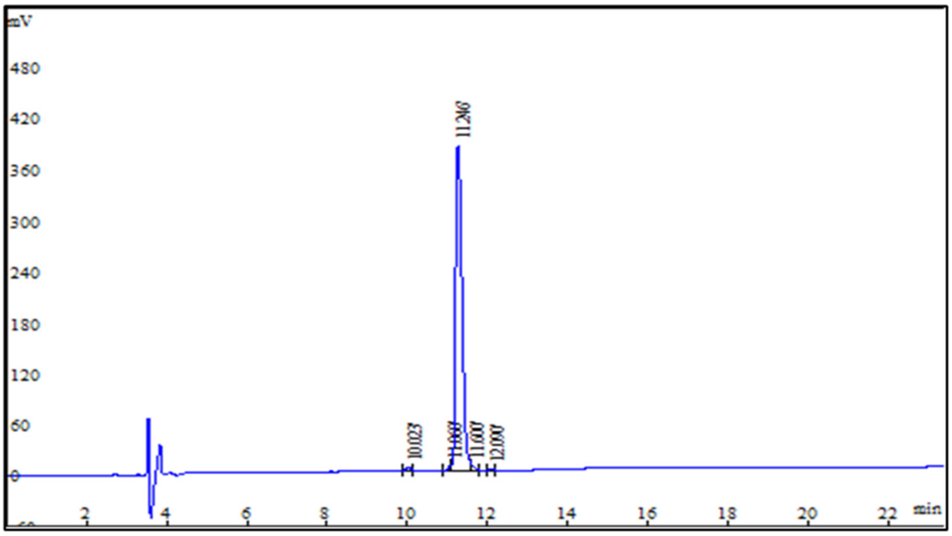

B

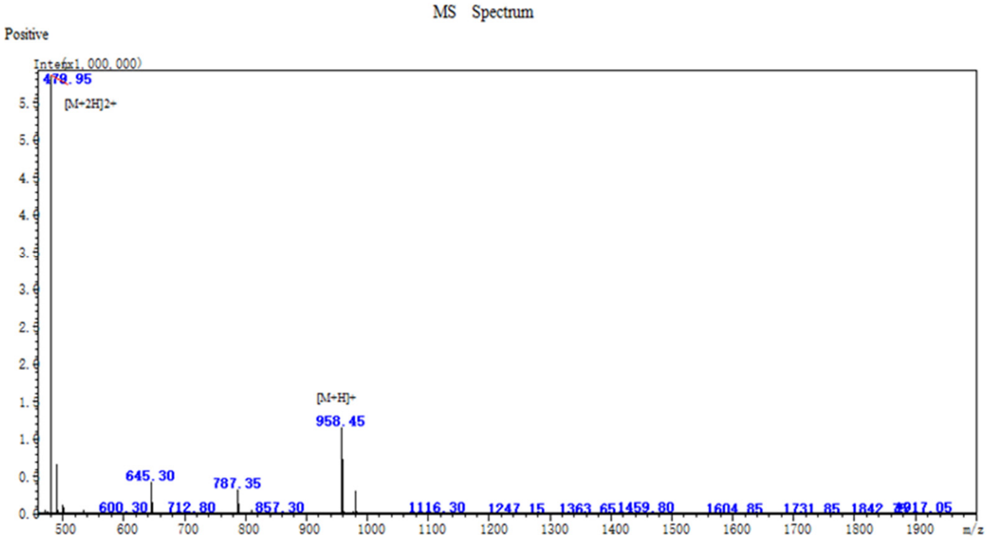

Figure S1. HPLC and LC-MS spectrum of synthetic peptide (LRGFGNPPT). A. HPLC spectrum of the synthetic peptide (LRGFGNPPT); B. LC-MS spectrum of the synthetic peptide (LRGFGNPPT).

Table S1. Physicochemical properties of LRGFGNPPT through ADMETlab 3.0.

Property	Value	Comment
Molecular weight	957.5 g/mol	Optimal: 100~600 g/mol
Volume	938.145 cm^3/mol	-
Density	1.021 g/cm^3	-
Number of hydrogen bond acceptors	25	Optimal: 0~12
Number of hydrogen bond donors	16	Optimal: 0~7
Number of rotatable bonds	35	Optimal: 0~11
Number of rings	3	Optimal: 0~6
Number of atoms in the biggest ring	6	Optimal: 0~18
Number of heteroatoms	25	Optimal: 1~15
Formal charge	0	Optimal: -4~4
Number of rigid bonds	27	Optimal: 0~30
Flexibility	1.296	-
Stereo centers	8	Optimal: ≤ 2
Topological polar surface area (TPSA)	403.76 Å^2	Optimal: 0~140 Å^2
Log of the aqueous solubility (LogS)	-2.039 log mol/L	Optimal: -4~0.5 log mol/L
Log of the octanol/water partition coefficient (LogP)	-0.778 log mol/L	Optimal: 0~3
LogP at physiological pH 7.4 (LogD)	-0.482 log mol/L	Optimal: 1~3

Table S2. ADMET properties of LRGFGNPPT through ADMETlab 3.0.

Property	Value	Decision
**Absorption**		
Caco-2 permeability	-6.639	🔴
MDCK permeability	-5.382	🔴
P-glycoprotein (Pgp)-inhibitor	0.013	🟢
P-glycoprotein (Pgp)-substrate	0.999	🔴
Human intestinal absorption (HIA)	0.999	🔴
20% bioavailability (F20%)	1.0	🔴
**Distribution**		
Plasma protein binding (PPB)	14.045	🟢
Volume distribution (VD)	-0.663	🔴
Blood-brain barrier (BBB) penetration	0.0	🟢
The fraction unbound in plasms (Fu)	93.625	🟢
**Metabolism**		
CYP1A2-inhibitor	0.0	🟢
CYP1A2-substrate	0.0	🟢
CYP2C19-inhibitor	0.0	🟢
CYP2C19-substrate	0.0	🟢
CYP2C9-inhibitor	0.0	🟢
CYP2C9-substrate	0.0	🟢
CYP2D6-inhibitor	0.0	🟢
CYP2D6-substrate	0.002	🟢
CYP3A4-inhibitor	0.0	🟢
CYP3A4-substrate	0.0	🟢
**Excretion**		
Plasma clearance	2.205	🟢
The half-life ($T_{1/2}$)	1.18	🟠
**Toxicity**		
hERG blockers	0.168	🟢
Human hepatotoxicity(H-HT)	0.344	🟠
Drug induced liver injury (DILI)	0.038	🟢
AMES toxicity	0.031	🟢
Rat oral acute toxicity	0.025	🟢
Maximum recommended daily dose (FDAMDD)	0.165	🟢
Skin sensitization	1.0	🔴
Carcinogenicity	0.003	🟢

Eye corrosion	0.0	●
Eye irritation	0.0	●
Respiratory toxicity	0.143	●

●: excellent, ●: medium, ●: bad

Table S3. Prediction of potential cleavage sites by various proteases for LRGFGNPPT using PeptideCutter.

Name of enzyme	No. of cleavages	Positions of cleavage sites
Chymotrypsin-high specificity (C-term to [FYW], not before P)	1	4
Chymotrypsin-low specificity (C-term to [FYWML], not before P)	2	1 4
Pepsin (pH 1.3)	2	1 3
Pepsin (pH > 2)	2	1 3
Trypsin	1	2
